# Incisura groove depth is associated with reduced rotational fibular displacement after syndesmotic injury: cadaveric validation of an automated three-dimensional incisura morphometric pipeline

**DOI:** 10.3389/fmed.2026.1870136

**Published:** 2026-06-26

**Authors:** Kareem Omran, Cesar de Cesar Netto, Sara Putnam, Alexander Sawatzke, Kevin Dibbern

**Affiliations:** 1 Department of Orthopaedic Surgery and Rehabilitation, University of Nebraska Medical Center, Omaha, NE, United States; 2 Department of Orthopaedic Surgery and Rehabilitation, Duke University Hospital, Durham, NC, United States

**Keywords:** ankle, ankle joint, ankle sprain, incisura fibularis, orthopeadic trauma, syndesmosis, syndesmotic injuries, toolbox

## Abstract

**Background:**

The tibial incisura provides the osseous constraint in which the fibula is seated at the distal tibiofibular syndesmosis. The morphology of this constraint varies substantially between individuals, making it plausible that incisura geometry modulates fibular displacement under a given injury pattern and the individual risk of injury under comparable mechanisms. Reproducible quantification of three-dimensional incisura morphometry is a prerequisite for testing these hypotheses. This study aimed to develop and validate an automated three-dimensional morphometric toolbox for the tibial incisura, and secondarily to test the resulting outputs in a controlled cadaveric model of syndesmotic injury.

**Methods:**

An automated MATLAB toolbox was developed to compute eight three-dimensional morphometric parameters of the incisura from CT-derived data. The toolbox was evaluated on 34 cadaveric tibiae from 17 matched specimen pairs for reproducibility across repeat acquisitions and for bilateral reliability. Under a cadaveric protocol of repeat scanning before and after complete four-ligament syndesmotic sectioning, outputs were correlated with fibular displacement magnitude from paired six-degree-of-freedom kinematics.

**Results:**

All eight toolbox outputs showed no significant difference between pre- and post-sectioning acquisitions of the same bone, demonstrating reproducibility across repeat CT acquisitions. Bilateral reliability was good for all eight outputs (ICC ≥ 0.75), with seven exceeding 0.80. Four of the six outputs tested against displacement were significantly negatively associated with the magnitude of external rotation: mean groove depth (*r* = −0.48, *p* = 0.004), maximum groove depth (*r* = −0.46, *p* = 0.006), the conventional single-level incisura floor depth (*r* = −0.44, *p* = 0.010), and total groove volume (*r* = −0.37, *p* = 0.030). The first principal component explained 85.4% of variance and was negatively associated with the magnitude of external rotation (*r* = −0.42, *p* = 0.015).

**Conclusion:**

The incisura metrics toolbox provides reproducible, bilaterally reliable, and fully automated three-dimensional morphometric characterization of the tibial incisura fibularis from CT-derived surface meshes, supplying the methodological foundation required for prospective clinical study of incisura morphology. As a secondary exploratory finding, larger osseous constraint envelopes were associated with reduced rotational fibular displacement under controlled cadaveric sectioning, while morphology did not modulate translational displacement, supporting the construct validity of the pipeline and indicating that incisura morphometry is a candidate input to future diagnostic and predictive injury models.

## 1 Introduction

The distal tibiofibular syndesmosis is a fibrous articulation between the distal tibia and fibula stabilized by four ligaments and by the osseous geometry of the tibial incisura fibularis. It transmits load between the tibia and fibula during weight-bearing and constrains fibular motion during ankle rotation. Disruption of this articulation accounts for approximately 20 % of operatively treated ankle fractures and represents a common injury in athletic populations, particularly in contact and cutting sports (1–5). Operative management aims to restore the native fibular position within the incisura, and although reduction techniques have been described to guide this process, malreduction rates remain substantial across reported series (4, 6–9). Malreduction of the distal fibula within the tibial incisura has been repeatedly linked to chronic pain, post-traumatic arthritis, persistent disability, and an elevated risk of secondary surgery, making accurate reduction one of the principal determinants of long-term recovery (4, 10, 11).

Reduction quality, in turn, partly depends on the bony anatomy of the distal tibia itself. Patients with shallow tibial incisurae have been shown to be overrepresented in ligamentous ankle injury cohorts and tend toward anterior fibular malreduction following surgical fixation, while deeper and more retroverted incisurae are associated with posterior malreduction (12–16). The convergence of these two observations, that morphology associates with both injury occurrence and the direction of post-surgical malreduction, suggests that the bony anatomy of the syndesmosis exerts a mechanical influence at multiple stages of injury and recovery. These patterns are reproducible across independent clinical series and raise the question of whether preoperative morphometric assessment could refine individualized surgical planning, and recent work has begun to identify discrete morphological phenotypes that influence stability after syndesmotic injury (14–19). The evidence is nonetheless almost exclusively observational, drawn from cohorts that differ in injury mechanism, sex, body mass, and loading history, and the contribution of incisura morphology itself has not been isolated under equivalent injury conditions (13).

This evidence base also rests on a measurement convention with its own limitations. The standard quantification of the tibial incisura is taken from a single axial CT slice at a fixed level 10 millimeters above the tibial plafond, from which linear and angular parameters such as incisura depth, width, facet angle, and anterior and posterior tubercle length are extracted (17, 20, 21). The convention is reproducible, easy to teach, and amenable to existing clinical CT workflows, which together explain its persistence across decades of imaging and surgical literature. Anatomical work has nonetheless shown that the deepest point of the notch does not consistently sit at the conventional level (15) and that the optimal measurement plane differs by sex (16), which means a single fixed cross-section can sample a different anatomical location in different individuals (21–26). Three-dimensional reconstructions have been used to extract the same linear and angular parameters in a volumetric visualization environment and have replicated the association between shallow incisura morphology and ligamentous ankle injury (12, 27). These approaches still sample the incisura at a single cross-sectional level, however, rather than across the whole superoinferior height, and depend on manual landmark placement that limits scalability. Whether three-dimensional measurement of the incisura captures information beyond what the single-level convention provides is unknown.

A reproducible, automated method for capturing the three-dimensional geometry of the incisura, in a way that integrates information across its full superoinferior extent rather than at any single fixed level, would address both the observational and methodological limitations described above and provide a quantitative morphometric foundation on which prospective study of incisura anatomy and its clinical correlates could be built. Accordingly, the primary aim of this study was to develop and validate an automated three-dimensional morphometric toolbox for the tibial incisura, evaluating its reproducibility across repeat acquisitions and its bilateral reliability between contralateral limbs. As a secondary, exploratory aim, the toolbox outputs were tested against fibular displacement in a controlled cadaveric model of syndesmotic injury, to demonstrate that the morphometric measurements behave in a biomechanically interpretable manner under standardized conditions.

## 2 Materials and methods

### 2.1 Study design and specimens

This cadaveric experimental study used 34 thawed fresh-frozen through-the-knee lower extremity specimens drawn from 17 donors (17 matched pairs; Table 1). Specimens were screened and confirmed to have no radiographic or clinical evidence of prior ankle fracture, prior ankle surgery, acute fracture, or gross deformity. The proximal portion of each specimen was prepared by removal of surrounding soft tissue while preserving the proximal tibiofibular joint. The proximal tibia was potted in polymethylmethacrylate and fixed in a plantigrade position within a radiolucent external loading frame. A calibrated loading mechanism applied 356 N of axial compression through the proximal tibial cut surface to approximate single-limb stance-phase ground reaction force. Each specimen underwent paired imaging in the intact state and after standardized sequential sectioning of the four primary syndesmotic ligaments. All sectioning was performed through a direct lateral fibular approach by a single fellowship-trained orthopedic surgeon. Ten complete plantar flexion and dorsiflexion manual range-of-motion cycles were performed after sectioning to mobilize the ankle joint prior to post-sectioning imaging.

**Table 1:** Anthropometric characteristics of the cadaveric cohort (17 donors, 34 limbs; 11 male and 6 female donors).

Characteristic	Mean ± SD	Median [range]
Age at death (years)	74.2 ± 8.3	77 [55–85]
Stature (cm)	174.8 ± 9.7	177.8 [157.5–190.5]
Body mass (kg)	84.3 ± 24.8	81.6 [42.6–155.1]
Body mass index (kg/m^2^)	27.3 ± 6.3	27.9 [15.2–42.7]

Data are summarized as mean ± standard deviation and as median with bracketed range. Body mass index was computed as body mass divided by the square of stature in meters.

### 2.2 Cadaveric imaging and segmentation

Cadaveric specimens were imaged in a dedicated extremity cone-beam weight-bearing CT system (HiRise; CurveBeam, Hatfield, PA) at an isotropic voxel size of 0.37 millimeters, with a metal artifact reduction algorithm applied during reconstruction. Left and right limbs were imaged sequentially within each session under identical scanner geometry and loading parameters, and the loaded configuration was maintained throughout each acquisition to ensure that all measured positional relationships reflected the loaded state.

Three-dimensional surface models of the tibia, fibula, and talus were generated from DICOM volumes using a semi-automated segmentation pipeline in Disior Bonelogic 2.0 (Paragon 28, Englewood, CO). The pipeline applied Hounsfield unit thresholding to isolate cortical and cancellous bone from surrounding soft tissue, followed by region-growing algorithms to produce closed volumetric bone masks for each structure. All segmentations then underwent review and manual refinement in 3D Slicer (version 5.6.2; Brigham and Women’s Hospital, Boston, MA) to correct any threshold-induced artifacts at bone boundaries. Quality control included visual inspection across orthogonal axial, coronal, and sagittal planes, confirmation of smooth surface topology free of stair-stepping artifact at the articular surfaces, and verification that the tibiofibular and tibiotalar joint spaces were resolved as distinct surfaces. Volumetric masks were then converted to triangulated surface meshes and exported in stereolithography file format for all downstream computational analyses. Right-sided tibiae were reflected across the sagittal plane prior to coordinate assignment to produce canonical left-sided configurations and to permit direct bilateral comparison in a single coordinate frame.

### 2.3 Incisura metrics toolbox architecture

**Figure 1:**
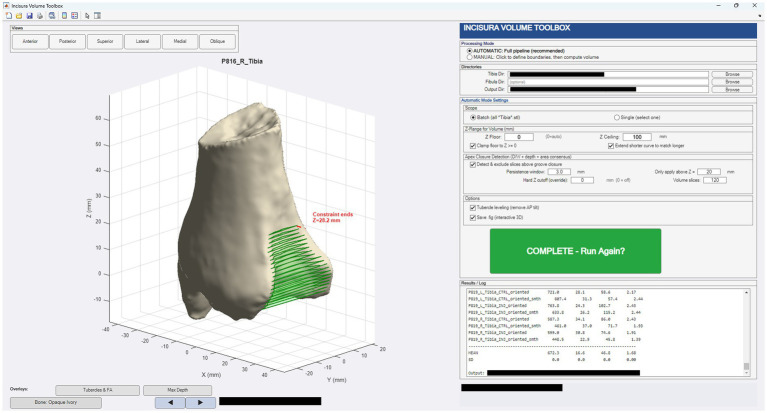
Incisura metrics toolbox graphical user interface in automatic mode on a representative right tibia. Following input of the segmented tibial mesh, the toolbox executes a fully deterministic pipeline from coordinate normalization through morphometric output without further user intervention. The right-hand panel exposes the parameters governing each stage of the pipeline: directories, batch or single-specimen scope, superoinferior integration range, apex closure detection, and tubercle leveling, together with a real-time results log of completed batch outputs. The central viewer renders the processed tibia with optional overlays of the computed metrics, with the constraint volume integration shown here as a stacked green wireframe of the cross-sectional contours sampled across the constraint envelope. View buttons (Anterior, Posterior, Superior, Lateral, Medial, Oblique) and overlay toggles (Tubercles & FA, Max Depth) at the bottom of the viewer permit interactive inspection of any computed output.

**Figure 2:**
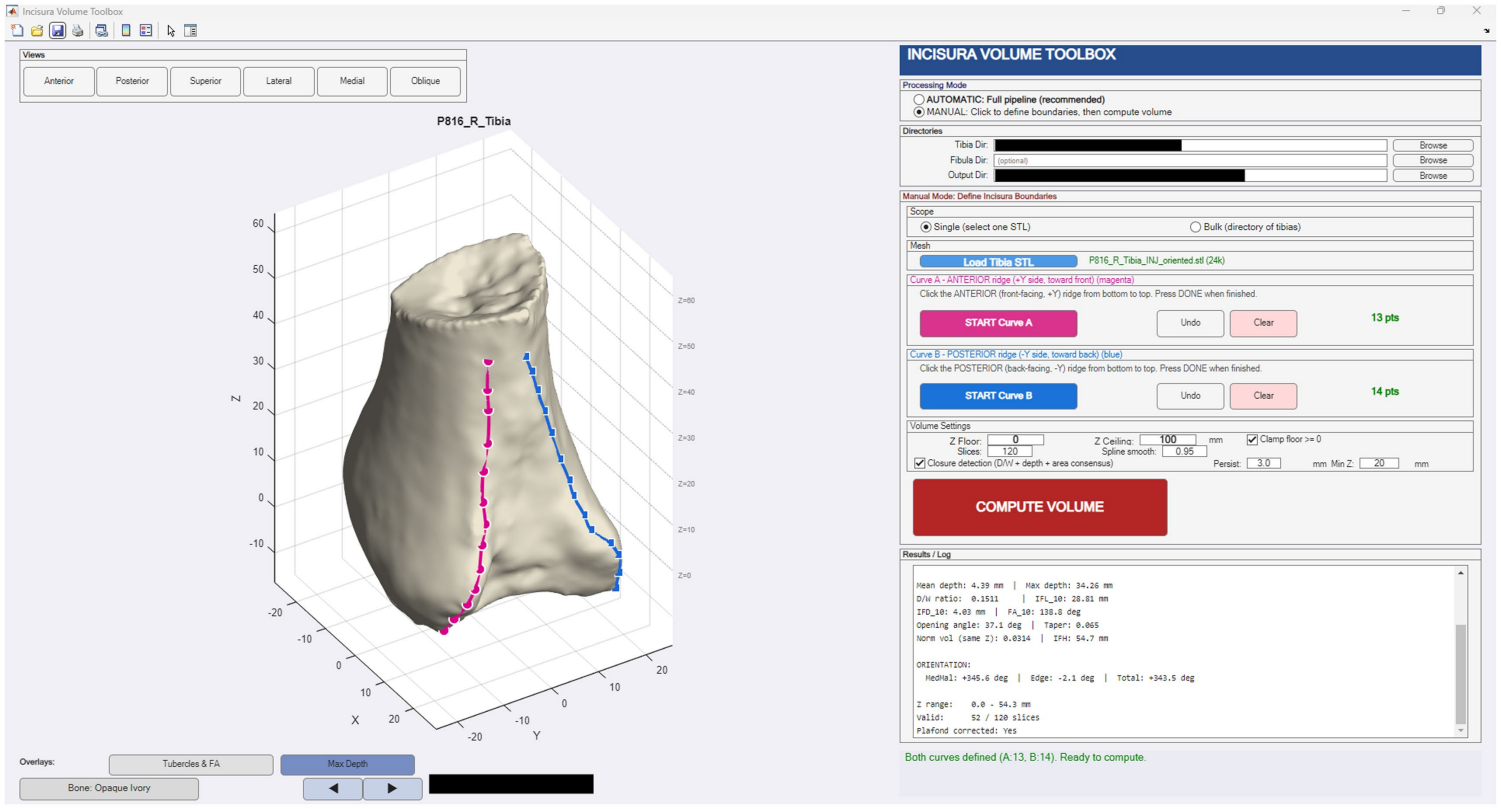
Interactive manual mode interface on the same right tibia, illustrating operator-controlled placement of the anterior and posterior boundary curves. Following user-specified curve placement on the rendered surface, all subsequent geometric computations, including coordinate normalization, cross-sectional area integration, and morphometric output extraction, proceed identically to the automatic mode pathway. The manual mode is intended for cases in which the operator elects to define ridge trajectories directly rather than rely on the template-guided automatic boundary detection.

The incisura metrics toolbox is implemented as a single MATLAB function (The MathWorks, Natick, MA) that launches an interactive graphical user interface with two processing modes. The Automatic mode executes a fully deterministic pipeline from mesh input to metric output without user intervention (Figure 1). The Manual mode provides interactive placement of the anterior and posterior boundary curves by the operator, after which all subsequent geometric computations proceed identically to the automatic pathway (Figure 2). Both modes output an identical metric set and support single-specimen and batch directory workflows. All analyses reported in the present work were produced using the Automatic mode, with manual verification of boundary curves confirming anatomically appropriate ridge trajectories in all 34 tibiae without requiring manual override. Boundary verification was performed by the lead author for each specimen. End-to-end processing time per specimen, from segmented mesh input to complete morphometric output, was approximately 10 to 20 s on a standard laptop workstation.

A dual-mesh architecture is maintained throughout processing. A Laplacian-smoothed detection mesh, produced by 30 smoothing iterations with a lambda coefficient of 0.4, is used exclusively for ridge identification and boundary curve extraction. All metric computations are performed on the unsmoothed native mesh to preserve the true osseous geometry of the individual specimen. This separation ensures that template-guided ridge detection is not confounded by surface noise while final metric outputs accurately reflect the anatomical dimensions of each bone.

#### 2.3.1 Coordinate system and scale normalization

A standardized anatomical coordinate system was established prior to morphometric measurement. Initial gross orientation was provided by the Automatic Anatomical Foot and Ankle Coordinate Toolbox (AAFACT) framework described by Peterson and colleagues, which aligns the tibia to a population-derived mean-shape template and defines the primary superoinferior, mediolateral, and anteroposterior axes (28). Two further geometric refinements were applied within the AAFACT-initialized frame. The medial malleolus centroid, identified from surface vertices with Z coordinate below minus 5 millimeters, was used to correct the mediolateral axis toward the true anatomical medial direction. The longest edge of the tibial plafond convex hull on the lateral side was then used to align the incisura face parallel to the anteroposterior axis, and a corrective rotation about the X axis removed any residual anteroposterior plafond tilt. No translational adjustment was applied, preserving the AAFACT convention in which the tibiotalar joint center lies at the coordinate origin.

Cross-sectional area–based scale normalization was then applied. The tibial cross-sectional area at 10 millimeters above the tibiotalar joint center was computed from the input mesh and compared against the corresponding area on the pre-computed mean-shape template. A scale factor was derived as the square root of the template-to-subject area ratio and applied uniformly to the specimen mesh before boundary detection. This template-scale alignment ensures that template-guided ridge detection performs consistently across the full range of tibial sizes present in the specimen cohort. All reported output metrics are expressed in true anatomical units by inverting the scale factor at the output stage, preserving dimensional fidelity in the final morphometric values.

#### 2.3.2 Anteroposterior midline and boundary curve extraction

The anteroposterior midline (Y_mid_) of the incisura at each superoinferior level was computed dynamically using a two-pass linear regression of the Y coordinates of tibial surface vertices within a fibula-proximity region of interest. The region of interest was identified by a k-d tree nearest-neighbor search between the subject tibial mesh and the paired fibular mesh, retaining the closest 20 percent of tibial vertices as the fibula-proximity cloud. A first-pass linear Y_mid_ fit was computed using a constant median Y value, boundary curves were constructed using this initial fit, and the Y_mid_ line was then refit from the resulting curves to produce a final midline that accounts for the approximately 30-degree anteroposterior twist of the incisura from inferior to superior levels.

Boundary curves defining the anterior ridge (corresponding to Chaput’s tubercle) and the posterior ridge (corresponding to Volkmann’s tubercle) were extracted through a two-stage process. In the first stage, pre-computed mean-shape template ridge curves were transferred to each specimen at corresponding Z levels and snapped to the nearest lateral mesh contour point within a 12-millimeter tolerance, filtered through a 5-millimeter corridor around the refined anterior and posterior detection planes to prevent snapping to anatomically implausible features. In the second stage, curvature-based vertex refinement relocated each snapped curve point to the local ridge maximum within a 3-millimeter search radius along the ordered mesh contour. Smoothed final curves were constructed from the refined points using cubic smoothing spline interpolation with 200 resampled curve points.

#### 2.3.3 Cross-sectional area and constraint envelope ceiling

Cross-sectional area at each superoinferior level was computed from a closed polygon defined by the anterior and posterior ridge anchors together with the intervening groove arc. The opening chord between the anchors closed the polygon medially, and the enclosed area was computed using the shoelace formula. A medial floor clipping step using the Sutherland–Hodgman polygon clipping algorithm removed any artifactual medial extension of the groove arc into the tibial shaft, which would otherwise inflate cross-sectional area estimates at levels where the incisura transitions toward the flat medial tibial surface (29).

The superoinferior integration range for volumetric computation was bounded inferiorly by the level at which the anterior and posterior boundary curves first converged and superiorly by a consensus closure ceiling. The closure ceiling was determined by four independent geometric criteria evaluated at each Z level. Method 1 flagged levels where the ratio of groove depth to chord width fell below 0.04, indicating the groove had flattened relative to its width. Method 2 flagged levels where the mean signed perpendicular distance from the chord to interior groove-arc points fell below 0.5 millimeters. Method 3 applied a cross-sectional area floor, flagging levels where the area fell below 25 percent of the cohort median area. Method 4 evaluated the concavity fraction of the groove contour, flagging levels where less than 50 percent of the interior arc was concave toward the groove axis. Each criterion produced a candidate closure Z, and the final ceiling was taken as the median of the candidate Z values when three or more criteria agreed, the minimum when exactly two agreed, and the single value when only one criterion fired. Constraint volume was computed by integrating cross-sectional area from the inferior boundary to the closure ceiling using the trapezoidal rule across 200 evenly spaced superoinferior levels.

#### 2.3.4 Primary morphometric outputs

**Figure 3:**
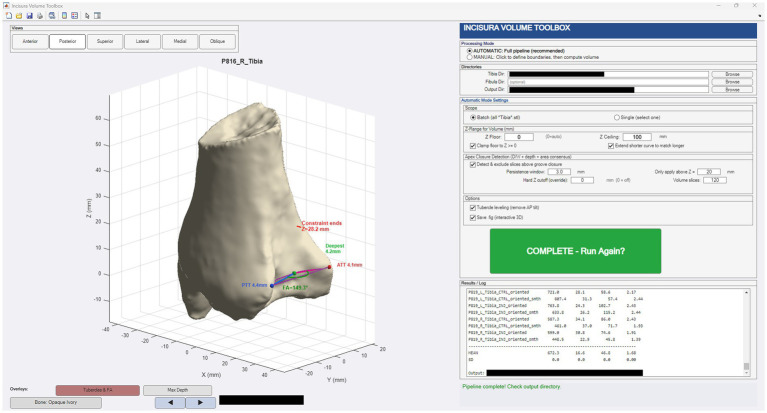
Primary toolbox outputs visualized on the same right tibia in the lateral oblique view. Annotated outputs include the deepest point of the groove (green marker, “Deepest”), maximum groove depth (4.22 mm at Z = 14 mm above the tibiotalar joint center), anterior tubercle prominence (red marker, ATT = 4.1 mm), posterior tubercle prominence (blue marker, PTT = 4.4 mm), the facet angle (FA = 149.3°) at the conventional 10-millimeter level, and the superior limit of the constraint envelope (red, “Constraint ends” at Z = 28.2 mm).

Eight scalar morphometric parameters were computed per specimen and specified prior to analysis to characterize the constraint envelope across four geometric dimensions, comprising volumetric constraint, groove depth, angular aperture, and anteroposterior tubercle geometry (Figure 3). The full set of parameter definitions is summarized in Table 2.

**Table 2:** Definitions of the eight morphometric parameters computed by the incisura metrics toolbox.

Parameter	Units	Definition
Constraint volume (FNV₁₀)	mm^3^	Trapezoidal integral of groove cross-sectional area from 10 mm above the tibiotalar joint center to the closure ceiling, representing the three-dimensional osseous constraint envelope available to the fibula across its full superoinferior engagement height.
Incisura floor depth (IFD₁₀)	mm	Maximum perpendicular distance from the anteroposterior chord to the deepest point of the groove contour at 10 mm above the tibiotalar joint center, measured in the mediolateral plane.
Facet angle (FA₁₀)	degrees	Angle subtended at the deepest groove point by vectors directed toward the anterior and posterior ridge tips at 10 mm above the tibiotalar joint center. Larger values indicate a shallower, more open notch configuration.
Maximum groove depth	mm	Maximum perpendicular mediolateral distance from the anteroposterior chord to the deepest point of the groove contour across all superoinferior levels within the constraint zone.
Mean groove depth	mm	Mean perpendicular mediolateral distance from the anteroposterior chord to the groove contour averaged across all superoinferior levels within the constraint zone.
Total groove volume	mm^3^	Total three-dimensional volume of the incisura groove integrated across the full superoinferior extent of the constraint zone. Complements FNV₁₀ by capturing the complete groove volume without restriction to levels above the plafond apex.
Anterior tubercle prominence	mm	Lateral X distance from the anterior tibial tubercle (Chaput’s tubercle) anchor to the deepest groove point at 10 mm above the tibiotalar joint center.
Posterior tubercle prominence	mm	Lateral X distance from the posterior tibial tubercle (Volkmann’s tubercle) anchor to the deepest groove point at 10 mm above the tibiotalar joint center.

### 2.4 Syndesmotic sectioning and six-degree-of-freedom displacement

Six-degree-of-freedom fibular displacement components were computed from bilateral CT registration of the intact and post-sectioning acquisitions using an ICP-based alignment framework with per-vertex correspondence weighting (30, 31). Three displacement components were selected for correlation with morphometric outputs on anatomical and biomechanical grounds. External rotation (Rz) was selected because it is the dominant displacement mode under physiological and injury loading conditions at the syndesmosis across cadaveric biomechanical studies (1, 3, 32), and because groove depth in the mediolateral plane defines the rotational arc available before osseous engagement. Anteroposterior translation (Ty) was selected because the anterior and posterior walls of the incisura directly bound the fibula in the anteroposterior plane, making this the anatomically anticipated outcome of constraint envelope morphometry. Lateral translation (Tx) was included as the displacement component targeted by conventional widening-based imaging criteria, despite the incisura providing no osseous barrier to mediolateral fibular escape, permitting direct testing of whether bony morphology modulates the component that clinical measurement currently relies upon.

### 2.5 Statistical analysis

Statistical analyses were performed in MATLAB R2025b (The MathWorks, Natick, MA). Two validation checks were performed prior to the primary correlation analysis. Pipeline and segmentation reproducibility was assessed by comparing morphometric outputs from intact and post-sectioning CT acquisitions of the same bone using paired Wilcoxon signed-rank testing of the eight toolbox outputs, on the assumption that syndesmotic ligament sectioning should not alter osseous geometry and that any pre- to post-sectioning difference would reflect combined CT acquisition and semi-automated segmentation variability rather than true anatomical change. Bilateral reliability across the 17 intact specimen pairs was quantified using two-way mixed-effects intraclass correlation coefficients with absolute agreement, interpreted according to the conventions of Koo and Li (33), under which values at or above 0.75 are considered to indicate good reliability. Systematic side-to-side bias was assessed using paired Wilcoxon signed-rank testing on left-versus-right values.

Bivariate Spearman rank correlations were then computed between six toolbox outputs, comprising constraint volume, single-level incisura floor depth, facet angle, mean groove depth, maximum groove depth, and total groove volume, and the magnitude of three fibular displacement components from the kinematic analysis, comprising external rotation, mediolateral translation, and anteroposterior translation. The Spearman test was selected over Pearson to accommodate the non-normal distribution of displacement values without imposing distributional assumptions. Principal component analysis of the eight standardized toolbox outputs was conducted to characterize inter-parameter co-variation across the constraint geometry, and the first principal component score was tested as a single additional predictor against the magnitude of each displacement component.

Multivariate modeling was not undertaken because the sample size of 34 limbs provides insufficient degrees of freedom relative to the number of candidate predictors, and because the morphometric parameters are geometrically related such that shared variance would produce unstable coefficient estimates. The analysis was treated as exploratory and hypothesis-generating, and formal correction for multiple comparisons was not applied; individual associations near the significance threshold are interpreted with appropriate caution. Statistical significance was accepted at *p* < 0.05 throughout.

## 3 Results

### 3.1 Descriptive morphometrics

The eight toolbox outputs across the 34 intact control tibiae demonstrated substantial inter-specimen variability, consistent with broad morphological heterogeneity of the tibial incisura previously reported in anatomical and imaging series (Table 3). Constraint volume (FNV₁₀) ranged from 114.7 to 880.0 mm^3^ (mean 402.4 ± 178.9 mm^3^). Incisura floor depth at 10 millimeters above the plafond apex ranged from 1.94 to 6.45 mm (mean 3.99 ± 1.10 mm). Facet angle ranged from 117.6 to 157.8 degrees (mean 141.0 ± 9.6 degrees). Mean groove depth ranged from 1.24 to 3.31 mm (mean 2.23 ± 0.52 mm), and maximum groove depth from 1.93 to 6.07 mm (mean 3.96 ± 1.03 mm). Total groove volume ranged from 347.3 to 1771.5 mm^3^ (mean 940.8 ± 337.2 mm^3^). Anterior tubercle prominence ranged from 1.74 to 6.62 mm (mean 3.64 ± 1.18 mm), and posterior tubercle prominence from 2.13 to 7.29 mm (mean 4.27 ± 1.12 mm). The superoinferior level at which the deepest point of the groove occurred ranged from 4.31 to 16.15 mm above the tibiotalar joint center (mean 12.04 ± 2.07 mm, median 12.28 mm). The conventional 10-millimeter sampling plane fell within 1 millimeter of the deepest point in 17.6% of specimens, while 50.0% of specimens had their deepest point within 1 millimeter of the 12-millimeter level.

**Table 3:** Descriptive statistics for the eight incisura metrics toolbox outputs across 34 intact control tibiae (17 matched specimen pairs).

Parameter	Mean ± SD	Median [Q1, Q3]	Range
Constraint volume (mm^3^)	402.4 ± 178.9	369.8 [270.0, 512.7]	114.7–880.0
Incisura floor depth (mm)	3.99 ± 1.10	3.88 [3.05, 4.47]	1.94–6.45
Facet angle (°)	141.0 ± 9.6	140.5 [135.3, 149.4]	117.6–157.8
Maximum groove depth (mm)	3.96 ± 1.03	3.94 [3.23, 4.44]	1.93–6.07
Mean groove depth (mm)	2.23 ± 0.52	2.16 [1.93, 2.56]	1.24–3.31
Total groove volume (mm^3^)	940.8 ± 337.2	923.5 [660.8, 1149.3]	347.3–1771.5
Anterior tubercle prominence (mm)	3.64 ± 1.18	3.54 [2.84, 3.97]	1.74–6.62
Posterior tubercle prominence (mm)	4.27 ± 1.12	4.27 [3.42, 4.92]	2.13–7.29
Height from plafond center of deepest point (mm)	12.04 ± 2.07	12.28 [11.16, 13.05]	4.31–16.15

SD, standard deviation; Q1, first quartile; Q3, third quartile.

### 3.2 Reproducibility across intact and post-sectioning acquisitions

All eight toolbox outputs showed no statistically significant difference between intact and post-sectioning CT acquisitions of the same bone on Wilcoxon signed-rank testing (Table 4). Wilcoxon *p*-values ranged from 0.209 (total groove volume) to 0.885 (mean groove depth), with no parameter approaching the conventional significance threshold. The absence of measurable change across independent scan sessions of identical osseous geometry confirms that the combined pipeline, including cone-beam CT acquisition, semi-automated segmentation, coordinate assignment, boundary detection, and metric computation, produces reproducible morphometric outputs at the level of within-specimen variability expected from repeated acquisition and segmentation.

**Table 4:** Reproducibility of the eight incisura metrics toolbox outputs across paired intact and post-sectioning CT acquisitions of the same specimens (*n* = 34 tibiae).

Parameter	Intact median	Post-sectioning median	Wilcoxon *p*
Constraint volume (FNV₁₀, mm^3^)	369.8	417.8	0.249
Incisura floor depth (IFD₁₀, mm)	3.88	4.17	0.521
Facet angle (FA₁₀, °)	140.5	139.0	0.791
Maximum groove depth (mm)	3.94	4.16	0.417
Mean groove depth (mm)	2.16	2.12	0.885
Total groove volume (mm^3^)	923.5	967.6	0.209
Anterior tubercle prominence (mm)	3.54	3.77	0.871
Posterior tubercle prominence (mm)	4.27	4.36	0.317

Wilcoxon signed-rank test for paired intact and post-sectioning acquisitions of the same specimen. No parameter showed a statistically significant pre- to post-sectioning difference. The absence of measurable change confirms that combined CT acquisition, semi-automated segmentation, coordinate assignment, and metric computation produce reproducible morphometric outputs.

### 3.3 Bilateral reliability

All eight toolbox outputs demonstrated intraclass correlation coefficients at or above 0.75 across the 17 intact specimen pairs, with no significant bilateral side difference on paired Wilcoxon signed-rank testing (Table 5). Constraint volume (FNV₁₀) demonstrated an ICC of 0.845 (bilateral *p* = 0.554), maximum groove depth an ICC of 0.880 (*p* = 0.177), and incisura floor depth (IFD₁₀) an ICC of 0.874 (*p* = 0.309). Total groove volume demonstrated an ICC of 0.867 (*p* = 0.493), facet angle an ICC of 0.843 (*p* = 0.084), mean groove depth an ICC of 0.756 (*p* = 0.493), anterior tubercle prominence an ICC of 0.810 (*p* = 0.084), and posterior tubercle prominence an ICC of 0.866 (*p* = 0.831). By the conventions of Koo and Li, all eight parameters exceeded the lower threshold for good reliability, with seven of the eight exceeding 0.80. Mean groove depth recorded the lowest coefficient at 0.756, at the lower bound of the good range. No parameter showed systematic side-to-side bias. The Z-location of the deepest groove point demonstrated comparable bilateral reliability (ICC = 0.797, *p* = 0.136), confirming that the longitudinal positioning of the deepest geometry is preserved between contralateral limbs.

**Table 5:** Bilateral reliability of the eight incisura metrics toolbox outputs across 17 intact specimen pairs.

Parameter	ICC (2-way mixed, absolute agreement)	Bilateral *p* (Wilcoxon)
Constraint volume (FNV₁₀)	0.845	0.554
Incisura floor depth (IFD₁₀)	0.874	0.309
Facet angle (FA₁₀)	0.843	0.084
Maximum groove depth	0.880	0.177
Mean groove depth	0.756	0.493
Total groove volume	0.867	0.493
Anterior tubercle prominence	0.810	0.084
Posterior tubercle prominence	0.866	0.831
Z-location of deepest point	0.797	0.136

All eight parameters demonstrated intraclass correlation coefficients (ICC) at or above 0.75 with no significant bilateral side difference. ICC thresholds from Koo and Li (33): 0.75–0.9 good, above 0.9 excellent.

### 3.4 Morphometric associations with the magnitude of fibular displacement

Bivariate Spearman correlations between the six toolbox outputs and the magnitude of each of the three fibular displacement components are presented in Table 6. Four of the six toolbox outputs were significantly negatively associated with the magnitude of external rotation following complete syndesmotic sectioning. Mean groove depth showed the strongest association (*r* = −0.480, *p* = 0.004), followed by maximum groove depth (*r* = −0.462, *p* = 0.006), the conventional single-level incisura floor depth (*r* = −0.438, *p* = 0.010), and total groove volume (*r* = −0.374, *p* = 0.030). Constraint volume and facet angle were not significantly associated with the magnitude of external rotation in this cohort. No toolbox output was significantly associated with the magnitude of mediolateral or anteroposterior translation. As no formal correction for multiple comparisons was applied, all associations are reported as exploratory; the association closest to the conventional significance threshold (total groove volume, *p* = 0.030) should be interpreted with particular caution and treated as hypothesis-generating.

**Table 6:** Bivariate spearman correlations between six incisura metrics toolbox outputs and the magnitude of three fibular displacement components from the kinematic analysis (*n* = 34 limbs).

Parameter	External rotation		Lateral translation		Anteroposterior translation	
	*r*	*p*	*r*	*p*	*r*	*p*
Constraint volume (FNV₁₀)	−0.263	0.133	+0.128	0.470	−0.330	0.058
Incisura floor depth (IFD₁₀)	−0.438	0.010 *	+0.035	0.844	−0.213	0.226
Facet angle (FA₁₀)	+0.308	0.077	−0.048	0.789	+0.108	0.542
Maximum groove depth	−0.462	0.006 **	−0.009	0.959	−0.209	0.235
Mean groove depth	−0.480	0.004 **	+0.101	0.567	−0.267	0.127
Total groove volume	−0.374	0.030 *	+0.021	0.907	−0.327	0.059
First principal component (PC1)	−0.416	0.015 *	+0.064	0.720	−0.261	0.135

*r* denotes the Spearman rank correlation coefficient and *p* denotes the two-tailed significance value. The first principal component (PC1) was derived from a principal component analysis of the eight standardized incisura metrics toolbox outputs and explained 85.4% of variance across the parameter set; its loading was approximately equal across the eight contributing outputs. * *p* < 0.05; ** *p* < 0.01. Bold means *p* < 0.05.

The first principal component of the eight standardized toolbox outputs explained 85.4% of variance across the eight parameters, with all eight outputs loading on the first component with comparable magnitude. The first principal component was significantly negatively associated with the magnitude of external rotation (*r* = −0.416, *p* = 0.015) but not with the magnitude of mediolateral or anteroposterior translation. The high proportion of variance explained by a single component, together with similar loadings across all eight outputs, indicates that the toolbox parameters reflect a common underlying construct of constraint envelope geometry rather than independent geometric features.

## 4 Discussion

The principal contribution of this study is the development and validation of an automated three-dimensional morphometric toolbox for the tibial incisura fibularis. As a secondary exploratory analysis, the toolbox outputs were tested against fibular displacement under a highly controlled cadaveric environment with complete four-ligament syndesmotic sectioning to confirm that the morphometric measurements behave in a biomechanically interpretable manner. Across 17 matched cadaveric specimen pairs, all eight toolbox outputs demonstrated reproducible behavior between independent CT acquisitions and bilateral reliability with intraclass correlation coefficients at or above 0.75, with no systematic side-to-side bias. Applied to the same specimens under standardized injury conditions, the four toolbox outputs that capture the depth and overall volume of the osseous constraint envelope were each significantly negatively associated with the magnitude of external rotation, with deeper grooves corresponding to less rotational fibular displacement. The first principal component of the eight outputs, which explained 85.4% of variance and represented the dominant axis of co-variation across the constraint geometry, was likewise associated with reduced rotational displacement. No morphometric parameter was significantly associated with the magnitude of mediolateral or anteroposterior translation. These findings provide the first direct evidence to the knowledge of the authors that incisura morphometry modulates the magnitude of rotational fibular displacement under controlled equivalent ligamentous disruption, with potentially significant diagnostic and operative implications for the management of syndesmotic injury.

The descriptive morphometric distribution of the present cohort aligns closely with the published anatomical literature. The mean facet angle, mean incisura floor depth, and mean tubercle prominence values all fall within ranges previously reported in cadaveric CT, dry bone, and MRI cohorts (17–22). The observed anteroposterior asymmetry, with the posterior facet and posterior tubercle consistently longer and more prominent than their anterior counterparts, recapitulates a pattern documented across populations and specimen types (18–20, 23, 30). The descriptive distributions from the present cohort therefore provide no evidence of systematic bias relative to the established morphometric literature, and the toolbox outputs can be interpreted against existing reference ranges without adjustment. The bilateral reliability data provide convergent validation against prior studies of incisura symmetry. Lepojärvi and colleagues found no significant asymmetry between uninjured ankles in single subjects with good inter- and intraobserver reliabilities, and Park and Kim confirmed the absence of significant side-to-side differences in bilateral ankle CT of uninjured volunteers (24, 31). The consequence of native bilateral symmetry for clinical diagnostics has been emphasized most directly by Kubik and colleagues, who demonstrated that unilateral CT assessment frequently produces anterior–posterior distance differences exceeding the standard malreduction threshold in uninjured ankles, and that a substantial proportion of native uninjured syndesmoses would be classified as malreduced by current diagnostic standards when assessed on bilateral CT (33). The toolbox’s bilateral reliability across the matched specimen pairs supports the validity of bilateral morphometric comparison as a quantitative framework and establishes the pipeline as suitable for deployment in studies that compare injured and uninjured contralateral ankles.

Against these validated morphometric results, the kinematic associations identified in the present analysis converge to a unified anatomical signal. All three depth parameters reached statistical significance against the magnitude of external rotation, with effect sizes in a comparable range. The three measurements quantify the same geometric relationship of perpendicular distance from the anteroposterior chord to the deepest groove point, differing only in whether the depth is sampled at one fixed superoinferior level or aggregated across the full superoinferior extent of the constraint zone. The convergence of the three measurements on a similar association with rotation suggests that under the morphometric range and sample size of this cadaveric cohort, the mechanical signal in the data is robust enough to be detected by either sampling strategy. In the present cohort, the deepest point of the groove sat a mean of 12.04 mm above the tibiotalar joint center on the plafond (SD 2.07, range 4.31 to 16.15) and the conventional 10-millimeter sampling plane fell within 1 millimeter of the true deepest point in only 17.6% of specimens, corroborating the documented offset between the conventional sampling level and the true geometric maximum of the groove. In larger or more morphologically heterogeneous cohorts, where this offset may translate into a greater fraction of mismeasured specimens, aggregation across the full constraint zone may capture mechanically meaningful geometry more consistently than a single fixed level.

The directional specificity of these findings is reinforced by the corresponding null observation along the mediolateral axis. The absence of any significant association between osseous morphometric parameters and the magnitude of lateral translation serves as a construct-validity check on the pipeline. The incisura is open laterally by anatomical definition, with its walls running anteriorly and posteriorly and its floor lying medially, but none of these surfaces creates a mechanical barrier to mediolateral fibular escape. Lateral fibular separation is resisted by the combined action of the interosseous ligament, the anterior inferior tibiofibular ligament, and the posterior inferior tibiofibular ligament, with the interosseous ligament demonstrating the highest tensile failure load of the three (34). Massri-Pugin and colleagues confirmed arthroscopically in a cadaveric series that no significant coronal diastasis occurs until all three syndesmotic ligaments are sectioned (35). Physiological lateral translation is small under normal loading conditions (36). In the present analysis, even complete four-ligament sectioning produced relatively modest lateral translation compared with the substantial external rotation magnitudes observed, and none of the bony morphometric parameters modulated the degree of this lateral escape. Beumer and colleagues demonstrated consistent results, showing that in the neutral position the largest displacements of the fibula following syndesmotic sectioning consisted of external rotation rather than lateral diastasis (37). A tool measuring osseous constraint geometry should produce a null association for a displacement component that osseous geometry does not resist, and the present toolbox produces that, in a manner that is clinically and biomechanically coherent with the ligamentous architecture of the coronal-plane syndesmotic constraint.

These results sit alongside a broader recent shift in syndesmotic assessment toward three-dimensional analysis. Three-dimensional volumetric approaches to syndesmotic assessment have emerged over the past decade and represent a substantial advance over single-slice measurement. Bhimani and colleagues demonstrated that three-dimensional tibiofibular joint space volume on weight-bearing CT discriminates syndesmotic instability more effectively than two-dimensional measurements (38). Ashkani-Esfahani and colleagues subsequently reported high sensitivity and specificity for subtle syndesmotic instability using volumetric assessment up to five centimeters above the plafond (32). De Cesar Netto and colleagues extended this work with a 3D distance mapping algorithm that detected significantly increased syndesmotic distances at all measured levels above the plafond following complete ligament sectioning under axial load, with diagnostic accuracy highest at the first one and three centimeters of the incisura (39). Furthermore, Peiffer and colleagues developed an automated pipeline using neural network segmentation and iterative closest point registration that produced bilateral 3D reference values with excellent manual-to-automated agreement (40). Each of these approaches, however, quantifies the position of the fibula relative to the tibia rather than the morphometry of the osseous groove itself. The joint-space volume captured by existing tools varies jointly with incisura geometry and fibular position and therefore conflates two distinct anatomical contributions to stability. The incisura metrics toolbox addresses this gap by providing volumetric characterization of the incisura as an osseous constraint envelope whose geometry reflects the passive resistance the bony articulation alone provides against fibular displacement, independent of the fibula’s instantaneous location.

Alongside these volumetric approaches, statistical shape modeling has emerged as the principal computational approach for characterizing population-level skeletal variation in the foot and ankle, and several recent studies have applied it to the distal tibiofibular joint and the broader syndesmotic complex (41–43). Statistical shape modeling builds a mean-shape template from a registered cohort and decomposes inter-specimen variation into orthogonal principal modes that, by construction, capture independent dimensions of geometric variability. However, statistical shape modeling does not, on its own, attach explicit anatomical meaning to the modes it extracts. The principal modes are conventionally interpreted by visual inspection of the rendered shape extremes, and the lack of clinical interpretation of the resulting modes of variation has been recognized as a recurring limitation of the technique. The incisura metrics toolbox may have utility in bridging this gap. Applied to the shapes generated at each end of a statistical shape model’s principal modes, the toolbox can produce explicit anatomical descriptors of how the PCA modes, or their extremes differ.

Clinically, these findings may carry implications for preoperative assessment and intraoperative reduction. The cadaveric design supports mechanistic inference about the constraint role of the bony incisura but cannot establish clinical risk; the implications outlined below are hence best interpreted as hypotheses for prospective study rather than as direct conclusions for current practice. Rotational fibular malreduction is invisible to conventional radiographic assessment, which detects coronal-plane lateral widening but not external rotation of the fibula within the incisura (44, 45). Existing operative imaging protocols rely heavily on the tibiofibular clear space and tibiofibular overlap on anteroposterior and mortise views to confirm reduction quality, and these measurements are insensitive to the rotational component of fibular position. Cadaveric work has demonstrated that the standard fluoroscopic indices remain within their normal range with up to 30 degrees of induced fibular external rotation and are unable to detect external rotational malreduction of this magnitude (44). Intraoperative reduction technique itself further influences fibular position within the incisura, with reduction clamp tine placement shown to systematically bias the resulting reduction direction, and the rotational component of the resulting fibular position is the most likely to escape conventional radiographic detection (46–48). Shallower incisurae and smaller constraint envelopes were associated in the present cohort with greater rotational fibular displacement under equivalent ligamentous disruption, identifying this morphometric phenotype as a candidate marker of heightened susceptibility to residual rotational malreduction following operative fixation. Bilateral CT assessment of the syndesmosis, using the contralateral uninjured ankle as a within-subject reference, has been advocated for several years to address the limitations of plain radiography, and morphometric stratification by preoperative incisura geometry could refine the indications for this advanced postoperative assessment (21, 49–51). Automated morphometric outputs of the kind produced by the toolbox take seconds to compute from a bilateral weight-bearing CT acquisition, and weight-bearing CT use in foot and ankle assessment has grown rapidly over the past decade, with multiple studies demonstrating superior diagnostic performance over conventional radiography and non-weight-bearing CT for syndesmotic instability detection (32, 52–54). Incorporating automated incisura morphometry into the same bilateral weight-bearing CT workflow may improve sensitivity for subtle syndesmotic injury where MRI is unavailable or has not yet been obtained.

Morphometric quantification of incisura geometry could, if these associations are reproduced clinically, help explain several clinical observations that current imaging alone cannot resolve. Patients with apparently equivalent injury mechanisms can experience markedly different injury severity, some patients develop chronic ankle instability after an isolated lateral ligament sprain while others do not, and some appear predisposed to syndesmotic malreduction following operative fixation (15, 18, 55, 56). Definitive linkage of incisura morphology to injury susceptibility, postoperative reduction quality, and long-term rehabilitation trajectory will require prospective clinical studies correlating preoperative morphometric outputs with intraoperative reduction quality, postoperative bilateral CT assessment, and patient-reported outcome measures at minimum two-year follow-up. The present cadaveric associations provide a biomechanically grounded rationale for that investigation and effect-size estimates to inform powering.

These findings however, should be interpreted in the context of several limitations. The sample of 34 limbs provides adequate power to detect moderate bivariate correlations but limits the ability to examine multivariate relationships among correlated morphometric parameters simultaneously; the eight toolbox outputs are geometrically related, with a single principal component explaining 85.4% of variance across them, and the independent contribution of any single parameter cannot be isolated in a bivariate framework where the underlying constraint envelope dimension dominates. The present analysis was designed as a validation of the toolbox and as exploratory identification of candidate morphometry–displacement associations to inform larger clinical studies powered for multivariate modeling, and the reported effect sizes provide preliminary estimates for such powering calculations. Statistical significance was accepted without formal correction for multiple comparisons; this is justified by the apriori specification of the displacement components and morphometric parameters on anatomical and biomechanical grounds rather than *post hoc* selection, but the possibility of inflated Type I error at borderline significance levels remains, and associations at the margins of conventional thresholds are interpreted with caution. The donor cohort consisted entirely of older decedents with no reported history of ankle injury, which may reflect a selection for protective morphological phenotypes; if individuals with shallower incisurae are at higher lifetime risk of ligamentous ankle injury as epidemiological data suggest (13), the present cohort may be skewed toward deeper and more constraining geometries relative to clinical populations in whom shallow morphology is overrepresented, potentially compressing the range of the predictor variables and attenuating observable correlation magnitudes. This is a generalizability limitation, as syndesmotic injury most commonly affects younger athletic populations rather than the older decedents from whom the present specimens were obtained, and the morphometric phenotypes most relevant to clinical injury may be underrepresented in this cohort. Future studies should address this directly by recruiting younger, more active, or injury-prone cohorts in whom the morphological distribution more closely reflects the clinical syndesmotic-injury population. Significant sex differences in incisura morphology have been documented in independent populations, but the present sample was insufficiently powered to stratify correlations by sex; the morphometry–displacement relationships may differ between male and female anatomy, and sex-stratified analysis in larger cohorts is warranted (24, 57). An anatomical limitation of the present scope is that constraint geometry at the syndesmosis is determined by both surfaces of the articulation, the tibial incisura and the apposed fibular notch, while the present toolbox characterizes only the tibial side. The fibular cross-sectional geometry, in particular the curvature and width of the fibular notch, modulates how the fibula engages the tibial groove and could plausibly explain residual displacement variance not accounted for by the tibial morphometry alone. Two specimens with identical tibial groove depth but different fibular geometry may experience different rotational constraint under equivalent injury. Extension of the toolbox to extract analogous morphometric parameters from the fibular surface, and to compute joint-level constraint metrics that combine both contributions, is a natural next step that we are actively pursuing. Finally, the cadaveric model does not replicate active neuromuscular stabilization or the time-dependent viscoelastic ligamentous behavior that modulates the *in vivo* response to syndesmotic injury, which necessarily limits the direct translation of the reported effect sizes to clinical loading. These limitations notwithstanding, the present work provides the first direct evidence to the knowledge of the authors that three-dimensional incisura morphometry is associated with the magnitude of fibular displacement under controlled equivalent ligamentous disruption, rather than serving as an epidemiological risk marker for injury occurrence at the population level.

In conclusion, the incisura metrics toolbox provides reproducible, bilaterally reliable, and fully automated three-dimensional morphometric characterization of the tibial incisura fibularis from weight-bearing CT-derived surface meshes. Applied within a controlled cadaveric model of complete syndesmotic sectioning, it provides evidence that three-dimensional incisura morphometry is associated with the magnitude of fibular displacement under controlled equivalent ligamentous disruption. Deeper grooves and greater constraint volumes were associated with reduced rotational fibular displacement, and the absence of any association along the mediolateral and anteroposterior translational axes is consistent with the open lateral architecture of the incisura and supports the construct validity of the pipeline. Whether these cadaveric associations translate into clinically meaningful differences in reduction quality, postoperative malreduction risk, or functional recovery remains to be established by prospective clinical study. The toolbox provides the reliable and reproducible morphometric quantification that such investigations will require.

## Data Availability

The raw data supporting the conclusions of this article will be made available by the authors, without undue reservation.

## Glossary

AAFACT: Automatic Anatomical Foot and Ankle Coordinate Toolbox
AITFL: anterior inferior tibiofibular ligament
CT: computed tomography
DOF: degrees of freedom
FA₁₀: facet angle at 10 millimeters above the tibiotalar joint center
FNV₁₀: fibular notch (constraint) volume from 10 millimeters above the tibiotalar joint center
ICC: intraclass correlation coefficient
ICP: iterative closest point
IFD₁₀: incisura floor depth at 10 millimeters above the tibiotalar joint center
IFL₁₀: incisura fibular length at 10 millimeters
IMT: Incisura Metrics Toolbox
IOL: interosseous ligament
IOM: interosseous membrane
PITFL: posterior inferior tibiofibular ligament
PROM: patient-reported outcome measure
ROI: region of interest
Rz: external rotation component
STL: stereolithography
Tx: lateral translation component
Ty: anteroposterior translation component
WBCT: weightbearing computed tomography
